# Interactions of IgG1 CH2 and CH3 Domains with FcRn

**DOI:** 10.3389/fimmu.2014.00146

**Published:** 2014-04-02

**Authors:** Tianlei Ying, Tina W. Ju, Yanping Wang, Ponraj Prabakaran, Dimiter S. Dimitrov

**Affiliations:** ^1^Protein Interactions Group, Cancer and Inflammation Program, National Cancer Institute, National Institutes of Health, Frederick, MD, USA; ^2^Basic Science Program, Leidos Biomedical Research, Inc., Frederick National Laboratory for Cancer Research, Frederick, MD, USA

**Keywords:** FcRn, antibody fragments, CH2 domain, CH3 domain, small-size

## Abstract

Antibody fragments are emerging as promising biopharmaceuticals because of their relatively small-size and other unique properties. However, when compared to full-size antibodies, most of the current antibody fragments of VH or VL display greatly reduced half-lives. A promising approach to overcome this problem is through the development of novel antibody fragments based on IgG Fc region, which contributes to the long half-life of IgG through its unique pH-dependent association with the neonatal Fc receptor (FcRn). The IgG Fc region comprises two CH2 and two CH3 domains. In this report, we present a comparative study of the FcRn binding capability of the CH2 and CH3 domains. The stability and aggregation resistance of these domains were also investigated and compared. We found that monomeric CH2 and CH3 domains exhibited the pH-dependent FcRn binding while the dimeric forms of CH2 and CH3 domains did not. Although all of these domains had high serum stability, they had aggregation tendencies as measured by dynamic light scattering. By providing a better understanding of the structure–activity relationship of the Fc fragment, these results guide further approaches to generate novel Fc-based small-size antibody fragments that possess pH-dependent FcRn binding capability, desired *in vivo* half-lives, and other favorable biophysical properties for their druggability.

## Introduction

Therapeutic antibodies are the fastest growing class of protein biopharmaceuticals, accounting for over half of therapeutic protein sales in 2010. Monoclonal antibodies (mAbs), in particular, are enjoying significant success in the clinic and have been used for the effective treatment of a number of diseases such as cancer and immune disorders (1). Currently, the vast majority of Food and Drug Administration (FDA)-approved therapeutic antibodies and those in clinical trials are full-size antibodies in IgG1 format (2). Due to their large size (~150 kDa), they exhibit poor penetration in tissues and limited binding to surface targets that could be accessible by molecules of smaller size. Therefore, antibody fragments – with a reduced size of 12–50 kDa – have the potential to overcome the fundamental limitations of full-size antibodies. Due to their smaller size, they could achieve enhanced tissue penetration and a wider range of possible targets, as well as require lower production costs (3–5). However, compared to IgG format antibodies, they display greatly reduced half-lives and as such have limited clinical potential.

IgG1 antibodies exhibit extremely long half-lives (up to 3 weeks in circulation) and broad biodistribution due to their unique pH-dependent association with the neonatal Fc receptor (FcRn). The FcRn is an intracellular receptor localized in the acidic compartments of endosomes (6). By fluid-phase endocytosis, circulating IgGs enter endosomes, the first step of the proteolytic degradation pathway. However, at the low pH of the endosome (pH 6.0), IgGs form a complex with FcRn and are then recycled to the cell surface. The higher physiological pH of the circulation (pH 7.4) triggers the release of IgGs from their complex with FcRn back into circulation (7). Therefore, a promising approach to improve half-life is the development of antibody fragments based on the IgG Fc region, which comprises the unit necessary for binding to FcRn. Fc-based fragments can be used to create Fc-fusion proteins and novel antibody scaffolds.

Fc-based fragments have proven to be successful in enhancing the half-life of many drug candidates that alone would have been in circulation for a scale of minutes to hours rather than weeks. These Fc fusions are composed of the effector domain of a macromolecule linked to the IgG Fc region, which confers a prolonged half-life and better production due to its interactions with FcRn and its relative stability, respectively (8). The first Fc-fusion etanercept (Enbrel™) was introduced in 1998 for the treatment of rheumatoid arthritis (9) and later a broad range of autoimmune diseases. As a tumor necrosis factor (TNF) inhibitor, Enbrel™ is composed of the TNF receptor fused to the Fc region of IgG1. Since its introduction, this class of therapeutic has grown rapidly, as the FDA has approved several Fc fusions for clinical use.

Fc-derived antibody fragments can also serve as novel antibody scaffolds that exhibit both effector functions and binding to FcRn (10–14). Moreover, the loop regions at the C-terminal of its CH3 regions can be engineered to form new antigen-binding sites (15). The Fc antigen-binding (Fcab) small-size antibody format developed by F-star has been shown to possess not only properties similar to that of full-size antibodies but also the added benefit of lower production costs. However, to further reduce the size of possible scaffolds, the IgG Fc region can be divided into two CH2 regions and two CH3 domains. These domains have the potential to be engineered into even smaller antibody fragments.

We have previously engineered monomeric CH2 (16), CH3 (11), and Fc-based fragments (12). Here, we present a comparative study of the FcRn binding capability of the monomeric and dimeric CH2 and CH3 domains. Both monomeric and dimeric CH2 and CH3 domains had high serum stability but also aggregation tendencies at different levels. Among these, we found that monomeric CH2 and CH3 domains exhibited the pH-dependent FcRn binding. Further, structural modeling studies of these domains in complex with FcRn were performed to understand the molecular basis of the FcRn binding. There results could be helpful to generate novel Fc region-based small-size antibody fragments that possess pH-dependent FcRn binding capability, desired *in vivo* half-lives, and other biophysical properties.

## Materials and Methods

### Antibody expression and purification

All antibody fragments, including Fc, mFc, dCH2, CH2, dCH3, mCH3, were expressed in *E. coli* HB2151, as described previously (11, 12, 17). The bacterial pellet was collected after centrifugation at 6,000 × *g* for 15 min, and resuspended in PBS (pH 7.4) containing 0.5 M NaCl and 0.5 milliunits/mL polymyxin B (Sigma-Aldrich). After 30 min incubation at room temperature, the bacterial pellet was subsequently clarified by centrifugation at 25,000 × *g* for 30 min at 4°C. The clarified supernatant was used for purification of antibody fragments by using HisTrap HP column (GE Healthcare) according to manufacturer’s protocols. Protein purity was determined by SDS-PAGE, and protein concentration was measured spectrophotometrically (NanoVue, GE Healthcare).

### Dynamic light scattering

For comparison of aggregation tendency, the antibody fragments were filtered through a 0.22-μm filter (Millipore, Bedford, MA, USA) and adjusted to the same concentration (0.25 mg/mL). Measurements were performed using a Zetasizer Nano ZS ZEN3600 (Malvern Instruments Limited, Westborough, MA, USA) to determine the size of protein particles. The samples (0.5 mL) were analyzed in polystyrene cuvettes with a path length of 10 mm at 25°C. Each sample was recorded three times with seven subruns of 10 s. The same samples were also used to run size exclusion chromatography (SEC) using FPLC AKTA BASIC pH/C system (GE Healthcare) with a Superdex 75 10/300 GL column. PBS (pH 7.4) was used as running buffer (flow rate 0.5 mL/min), and eluting proteins were monitored at 280 nm. The molecular mass standards used were ribonuclease A (13.7 kDa), chymotrypsinogen A (25 kDa), ovalbumin (44 kDa), bovine serum albumin (67 kDa), and aldolase (158 kDa), as described previously.

### Serum stability

The stabilities of dCH2, CH2, dCH3, and mCH3 in serum were evaluated for up to 12 days. Normal human serum was collected from healthy human donors approved by the NCI-Frederick Research Donor Program. Aliquots (0.1 mL) of each antibody fragment solution (12 μM) were mixed with 0.5 mL of normal human serum to give a final concentration of 2 μM. The mixture was passed through a 0.22-μm filter (Millipore, Bedford, MA, USA), and then incubated at 37°C. An aliquot was taken out at each time point and immediately stored at −80°C. After 10 days incubation, Western blots were performed to check the serum stability. Samples were electrophoresed through SDS-PAGE and transferred onto 0.2 μm nitrocellulose membranes (Bio-Rad). After transfer, membranes were blocked with 3% milk in PBS for 1 h and then incubated with anti-His Tag monoclonal antibody (ABM, Vancouver, BC, Canada). Membranes were washed and then incubated with anti-mouse IgG–alkaline phosphatase antibody (Sigma-Aldrich). After washing, immune complexes were detected by reaction with BCIP/NBT substrate solution (Sigma-Aldrich).

### FcRn binding measured by ELISA

Recombinant human single-chain soluble FcRn (sFcRn), containing both β and α chains in a 1:1 molar ratio, was expressed in mammalian cells and purified as described previously (18). ELISA wells were coated with sFcRn at 50 ng per well in PBS (pH 7.4) overnight at 4°C, and blocked with protein-free blocking buffer (Thermo Scientific) at 37°C for 2 h. Twofold serially diluted protein, prepared with PBS containing 0.2% BSA, pH 6.0 or PBS containing 0.2% BSA, pH 7.4, was added and incubated at 37°C for 1.5 h. The plates were washed with PBST (PBS plus 0.05% Tween-20), pH 6.0 or pH 7.4, and horseradish peroxidase (HRP)-conjugated anti-FLAG tag antibody (Sigma-Aldrich) in PBS (pH 6.0 or 7.4) was incubated with wells for 45 min at 37°C. After extensive washes with PBST (pH 6.0 or 7.4), the binding was detected by the addition of ABTS substrate (Roche, Indianapolis, IN, USA), and monitored at 405 nm.

### Homology-modeling and energy minimization

The three dimensional (3D) models of human FcRn, mFc, mCH2, and mCH3 were predicted by using the structural components from the crystal structure of rat FcRn–Fc complex (19) (PBD entry: 1I1A) as templates. Homology-modeling was carried out using SWISS-MODEL workspace (20), a fully automated protein structure homology-modeling server accessible via the ExPASy web server. After the generation of initial coordinates, each of the resulting models was refined by energy minimization using “Not just A Molecular Dynamics” (NAMD) program (21). The structures were solvated in a spherical box of water molecules using VMD program, with a minimal distance of 3 Å from the protein to the box boundary of sphere, and all the histidine residues were assigned as protonated. Systems were first minimized 5,000 steps, followed by a molecular dynamics simulation with the temperature gradually dropped from 350 to 100 K. The system was then re-minimized for 50,000 steps with conjugate gradient method. The non-bonded interaction cutoff distance was set to be 9 Å. The final minimized structures were taken for molecular docking.

### Molecular docking and interface analysis

Docking of FcRn and Fc-based fragments were performed using the ZDOCK module (22) on Discovery Studio 2.5 (Accelrys Inc.), which uses a grid-based representation of two proteins and a 3D fast Fourier transform (FFT) to effectively explore the rigid body search space of docking positions. The top ranked complex prediction, formed by the receptor FcRn and the ligand mFc, CH2, or mCH3, were solvated and subjected to further dynamic simulations run for 250 ps, with other parameters the same as above, to allow adjustment of the positions of side-chains and helices. The final minimized complex structures were analyzed by using VMD software (23) and PDBePISA (24), an interactive tool for the exploration of macromolecular interfaces at the European Bioinformatics Institute.

## Results and Discussion

### Aggregation propensities of Fc, mFc, dCH2, CH2, dCH3, and mCH3 fragments

**Figure 1:**
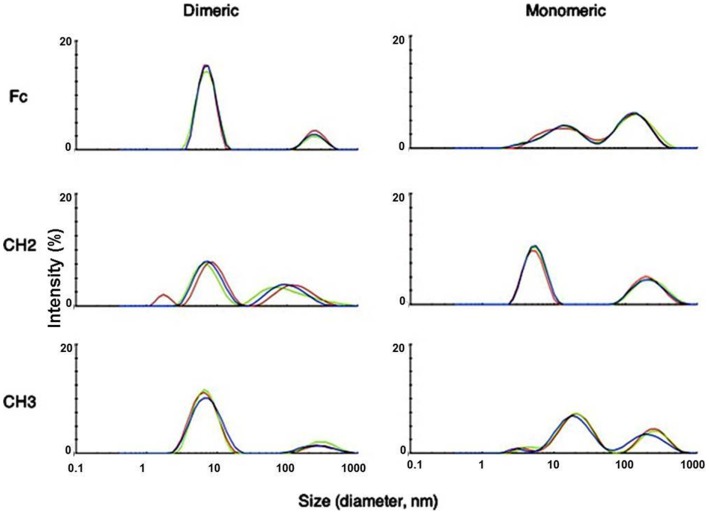
**Measurement of aggregation for IgG1 Fc fragments at 37°C by DLS with the *X*-axis denoting protein particle size (diameter, nanometer) and the *Y*-axis intensity (%)**. Three different colors depict three different measurements of the same fragments. Major peaks observed at different sizes indicated possible oligomer formation in these fragments after purification.

We used dynamic light scattering (DLS) to measure the aggregation tendencies of Fc-based fragments consisting of Fc, CH2, and CH3 in monomeric and dimeric forms at 37°C. We observed that both dimeric Fc and dCH3 had a major peak at a smaller size followed by a minor peak at a larger size indicating the predominance of monomeric species with the lesser extent of soluble oligomers (Figure 1). In contrast, the peaks at the larger size were increased for mFc, mCH3, dCH2, and CH2 reflecting the formation of large soluble oligomers of these fragments. Interestingly, the supernatants from most of the fragments were still monomeric according to the results obtained by using SEC (data not shown). Such findings indicate that the formation of the soluble oligomers may be reversible and the oligomers disassociate to monomers in diluted condition when running SEC.

### Serum stability of dCH2, mCH2, dCH3, and CH3 fragments

**Figure 2:**
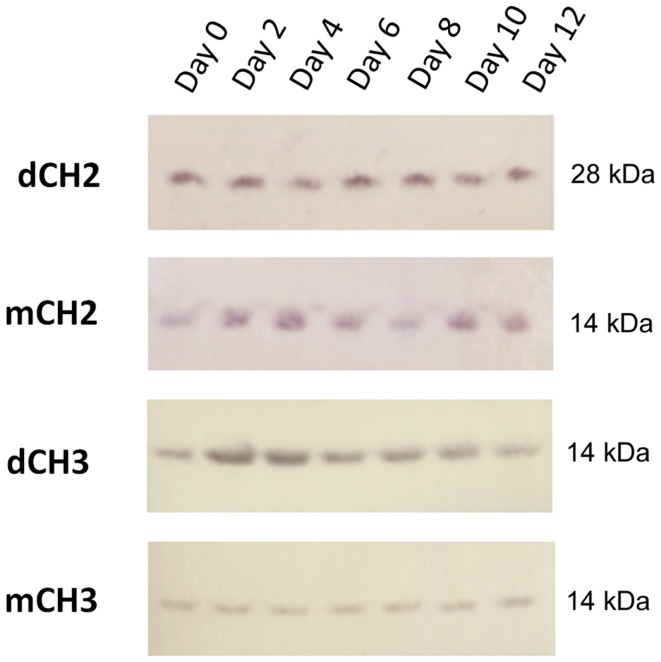
**Western blot–direct serum analysis of dCH2, CH2, dCH3, and mCH3 measured up to 12 days**.

We evaluated the stability of dCH2, mCH2, dCH3, and CH3 fragments in serum. Samples were incubated with human serum at 37°C, and an aliquot was taken out at each time point, for every other day, and stored at −80°C before Western blot (Figure 2) analyses for up to 12 days. These data suggest that dimeric and monomeric fragments of CH2 and CH3 domains have high serum stability.

### Monomeric CH2 and CH3 fragments bind to FcRn

**Figure 3:**
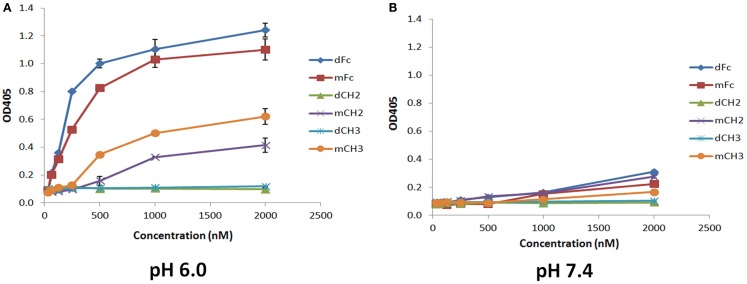
**Binding of IgG1 Fc-dimeric/monomeric fragments, dFc/mFc, mFc, dCH2/mCH2, and dCH3/mCH3, to hFcRn at pH 6.0 (A) and pH 7.4 (B) measured by ELISA**. The experimental points are represented as mean ± SD (*n* = 3).

To test whether the dCH2, mCH2, dCH3, and mCH3 fragments are functional and behave in a manner similar to wild-type or mFc, ELISA binding experiments were carried out to validate their pH-dependent binding to FcRn. The binding was performed under pH 6.0 and pH 7.4, respectively, as described in the experimental method section. As shown in Figure 3, the mCH2 and mCH3 fragments displayed a behavior similar to that of the wild-type or mFc. At pH 6.0, the mCH2 and mCH3 fragments showed detectable binding to FcRn (Figure 3A) while dCH2 and dCH3 did not show any binding to FcRn. At pH 7.4, neither wild-type Fc nor the fragments showed detectable binding to FcRn (Figure 3B). These results suggested that the mCH2 and mCH3 maintained characteristic pH-dependent FcRn binding while the dimeric forms of CH2 and CH3 fragments did not.

### Molecular modeling and docking studies of FcRn complexes with monomeric Fc, CH2, and CH3 domains

**Figure 4:**
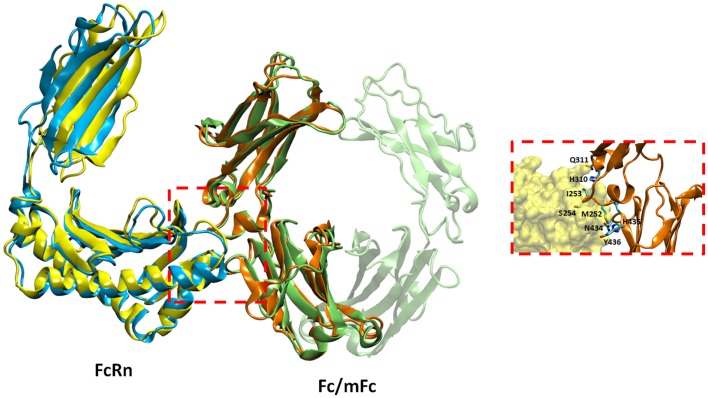
**Docked model of hFcRn–mFc complex as superimposed with the known rat FcRn–Fc complex structure**. The inset shows a close-up view of the molecular interface of hFcRn (yellow surface) and mFc (orange ribbons) with potential binding residues (as sticks).

To understand the details of potential molecular interactions underlying FcRn binding with the Fc fragments mFc, mCH2, and mCH3, homology-based molecular modeling of hFcRn and the Fc fragments was carried out. We then used these models for docking of hFcRn complexes with mFc, mCH2, and mCH3 fragments to compare the possible difference between the complexes in hFcRn binding and properties. Figure 4 depicts the docked model of hFcRn–mFc/Fc complex as superimposed with the known rat FcRn–Fc complex structure. The structure of rat FcRn was used as a template for building the structure of its human counterpart. The root-mean-square deviation (RMSD) values for Cα atoms were calculated as 1.39 Å between the aligned human and rat FcRn components of modeled and known template structures. The inset of Figure 4 shows a close-up view highlighting the molecular interface of the hFcRn (yellow surface) and mFc (orange ribbons) complex, along with the amino acid residues of mFc (in sticks) that could potentially make contact with the hFcRn. The FcRn–mFc interface accounts for a total of 1,261 Å^2^ buried surface area and involves specific interactions such as hydrogen bonds, salt bridges, and hydrophobic interactions. These interactions occur mostly at the hinge region between CH2 and CH3 domains of mFc, which contains several histidine and charged residues (Table 1) that resemble the rat FcRn/heterodimeric Fc complex (19).

**Table 1:** **Buried surface areas (BSA, Å^2^) and hydrogen bonds in human FcRn docked complexes with mFc, CH2, and CH3 fragments**.

FcRn–mFc DOCKED COMPLEX							
FcRn	BSA (Å)	mFc	BSA (Å)	FcRn		mFc	Distance (Å)
LEU 108	34.94	MET 252	30.29	GLU 111[OE1]	…	GLN 311[N]	3.13
GLU 111	110.41	ILE 253	136.06	GLU 111[OE2]	…	HIS 310[N]	2.85
TRP 127	124.70	SER 254	105.88	GLU 111[OE2]	…	HIS 310[ND1]	2.77
PRO 128	98.42	ARG 255	49.12	ASP 126[O]	…	HIS 435[N]	3.81
GLU 129	46.09	LEU 309	27.19	PRO 128[O]	…	TYR 436[OH]	3.16
LEU 131	59.09	HIS 310	22.16	GLU 129[OE2]	…	ILE 253[N]	3.15
		LEU 314	12.89	GLU 129[OE2]	…	SER 254[N]	3.26
		ASN 434	91.51				
		HIS 435	38.90				
		TYR 436	60.64				

**FcRn–CH2 DOCKED COMPLEX**							
**FcRn**	**BSA (Å)**	**CH2**	**BSA (Å)**	**FcRn**		**CH2**	**Distance (Å)**
TYR 84	15.30	LEU 251	22.20	GLU 112[N]	…	GLN 311[OE1]	3.80
LEU 108	28.13	MET 252	38.86	GLU 111[OE2]	…	HIS 310[ND1]	2.70
GLU 111	96.47	ILE 253	122.93	GLU 111[OE2]	…	HIS 310[N]	3.00
GLU 112	36.61	SER 254	77.13	GLU 129[OE1]	…	ILE 253[N]	3.13
TRP 127	130.11	LEU 309	20.60	GLU 129[OE2]	…	SER 254[N]	3.65
PRO 128	29.30	HIS 310	31.51				
GLU 129	43.40	GLN 311	57.87				
		LEU 314	23.87				

**FcRn–CH3 DOCKED COMPLEX**							
**FcRn**	**BSA (Å)**	**CH3**	**BSA (Å)**	**FcRn**		**CH3**	**Distance (Å)**
ASP 126	91.07	MET 428	25.31	ASP 126[OD1]	…	HIS 433[ND1]	2.91
TRP 127	27.44	HIS 433	16.50	ASP 126[OD1]	…	ASN 434[N]	2.69
PRO 128	51.41	ASN 434	65.11				
LEU 131	67.82	HIS 435	68.70				
LEU 263	22.02	TYR 436	53.79				

**Figure 5:**
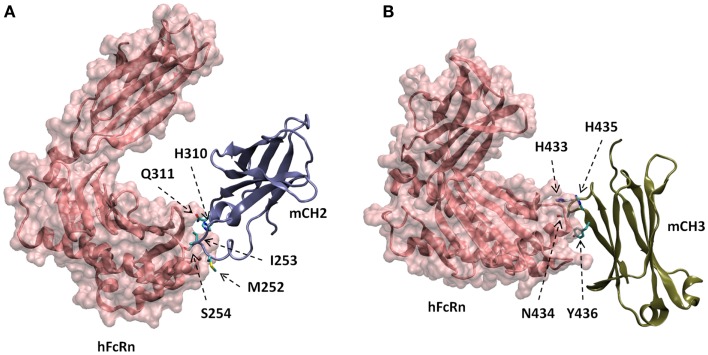
**Docked models of hFcRn complexes with mCH2 (A) and mCH3 (B), showing with putative amino acids in mCH2 and mCH3 domains involved in the interactions with hFcRn**.

The docked models of hFcRn complexes with mCH2 and mCH3 were illustrated in Figure 5. The overall contribution to the binding interface areas of these model complexes are much smaller, with 807 and 584 Å^2^ of buried surface areas at the interface of hFcRn–mCH2 and hFcRn–mCH3, respectively, compared to the FcRn–mFc model complex. However, some of the critical intermolecular salt bridges were found between hFcRn and Fc fragment – mFc, mCH2, and mCH3 – docking model complexes. In particular, His310 in CH2 and His433 in CH3 could interact with the acidic residues Glu112 and Asp126 of FcRn, respectively. Similar salt bridges between Fc histidine residues, His310 and His435, and FcRn acidic residues, Glu111/Asp126, respectively, were observed in the hFcRn–mFc model complex. The putative titrating salt bridges that involve acidic–histidine residue pairs at the interfaces of hFcRn–mFc, hFcRn–mCH2, and hFcRn–mCH3 complexes might be ascribed to the mechanism of pH-dependent FcRn binding. The different intermolecular interactions predicted in the docked model hFcRn complexes of mFc, mCH2, and mCH3 fragments may have resulted in their differential binding properties (Figure 3).

One last consideration is that the preferential binding of hFcRn to mCH2/mCH3 over their dimeric counterparts, dCH2/dCH3, as seen in Figure 3, may be due to the exposure of additional residues after the monomerization of the dimers. It may not be due to the size-dependent effect alone since the hFcRn binding to dFc and mFc were much stronger. However, it should be noted that these data were obtained only using an ELISA assay where the protein conformation may not completely mimic the conformation in their native state as expressed at the cell surface. Further studies using natively expressed proteins are needed to validate these findings.

## Conclusion

In summary, this study suggests that mCH2 and mCH3 could be potentially applicable in the engineering and development of novel biopharmaceuticals due to their high serum stability and pH-dependent FcRn binding. We found that while both monomeric and dimeric CH2 and CH3 domains had high serum stability and exhibited varying aggregation tendencies, only mCH2 and mCH3 bound to FcRn at pH 6.0. None of the antibody fragments bound to FcRn at pH 7.4. Our molecular modeling and docking studies suggest that this preferential binding of mCH2 and mCH3 to FcRn may be due to additional exposed residues from their monomerization. The smaller size and other described properties of mCH2 and mCH3 would overcome the fundamental problem of full-size antibodies, resulting in enhanced tissue penetration and providing access to a wide range of targets that are sterically occluded.

## Conflict of Interest Statement

The authors declare that the research was conducted in the absence of any commercial or financial relationships that could be construed as a potential conflict of interest.
